# May Bacterial Infections Trigger Bullous Pemphigoid? Case Report and Review of Literature

**DOI:** 10.3390/microorganisms9061235

**Published:** 2021-06-07

**Authors:** Michela Ileen Biondo, Chiara Fiorentino, Severino Persechino, Antonella Tammaro, Angela Koverech, Armando Bartolazzi, Salvatore Raffa, Marco Canzoni, Andrea Picchianti-Diamanti, Roberta Di Rosa, Giovanni Di Zenzo, Enrico Scala, Giorgia Meneguzzi, Claudia Ferlito, Milica Markovic, Sara Caporuscio, Maria Laura Sorgi, Simonetta Salemi, Bruno Laganà

**Affiliations:** 1 Dipartimento di Medicina Clinica e Molecolare, Sapienza, Università di Roma, AOU S. Andrea, 00189 Roma, Italy; biondo_michela@yahoo.it (M.I.B.); chiara.fiorentino@hotmail.it (C.F.); angelakoverech@gmail.com (A.K.); armando.bartolazzi@ospedalesantandrea.it (A.B.); salvatore.raffa@uniroma1.it (S.R.); canzonimarco@gmail.com (M.C.); andrea.picchiantidiamanti@uniroma1.it (A.P.-D.); roberta.dirosa@uniroma1.it (R.D.R.); giorgia.meneguzzi@hotmail.it (G.M.); clau.ferlito@gmail.com (C.F.); minamarkovic@hotmail.com (M.M.); sara.caporuscio1@gmail.com (S.C.); marialaura.sorgi@uniroma1.it (M.L.S.); 2 UOD di Dermatologia, Sapienza, Università di Roma, AOU S. Andrea, 00189 Roma, Italy; severino.persechino@uniroma1.it (S.P.); antonella.tammaro@uniroma1.it (A.T.); 3 Istituto Dermopatico dell’Immacolata, 00167 Roma, Italy; g.dizenzo@idi.it (G.D.Z.); e.scala@idi.it (E.S.)

**Keywords:** bullous pemphigoid (BP), infections, autoimmunity, IVIg

## Abstract

Bullous pemphigoid (BP) is an autoimmune blistering skin disease, mainly observed in the elderly. Infections have been suggested as possible disease triggers. However, infections may even heavily influence the disease clinical course and mortality. A 75-year-old woman was admitted to hospital for severe erythematosus blistering disease, accompanied by hyper-eosinophilia and hyper-IgE. The culture of bullous fluid was positive for *Enterococcus faecalis*, the blood culture was positive for *Staphylococcus aureus*, and the urine culture was positive for *Proteus mirabilis* and *Escherichia coli.* Moreover, circulating anti-BP180 IgG was present and the histopathological/ultrastructural examination of a lesional skin biopsy was compatible with BP. High eosinophil levels (up to 3170/µL) were found throughout the clinical course, while values below 1000/µL were associated with clinical improvement. The total IgE was 1273 IU/mL, and specific anti-G/V-penicillin/ampicillin IgE antibodies were positive. The patient had a complete clinical recovery in two months with methyl-prednisolone (40 then 20 mg/day) and low-dose azathioprine (50 mg/day) as a steroid-sparing agent. The steroid treatment was tapered until interruption during a one-year period and intravenous immunoglobulins have been administered for three years in order for azathioprine to also be interrupted. The patient stopped any treatment five years ago and, in this period, has always been in good health. In this case, the contemporaneous onset of different bacterial infections and BP is suggestive of bacterial infections acting as BP trigger(s), with allergic and autoimmune pathways contributing to the disease pathogenesis.

## 1. Introduction

Bullous pemphigoid (BP) is the most frequent of the rare autoimmune bullous skin diseases [1]. It is characterized by subepidermal blistering caused by autoantibodies directed against the hemidesmosomal proteins BP180 and BP230 [2,3]. Its incidence in Europe ranges from 4.47 to 13.4/1,000,000 person-years [4,5], occurring preferentially in the elderly, without a net predilection for sex [6]. BP is more frequently observed after the seventh decade, but particularly after the ninth decade of age [7]. The mortality is lower in the USA, but in Europe, it may be as high as 19–40% in the first year after flare [8]. A significant association with the major histocompatibility complex class II allele HLA-DQB1* 0301 was already observed in 1996 [9].

Several conditions have been considered risk factors for BP development: patients with BP are more likely to have various neurological diseases, including stroke, epilepsy, multiple sclerosis, Parkinson’s disease, dementia, and bedridden condition [10]. The association of BP with malignancy is controversial [11,12]. Despite the inability of a trigger to be identified in the large majority of BP cases [1], known agents/events involved in triggering BP include drugs, trauma, burns, ultraviolet radiation, radiotherapy, and vaccinations, particularly against influenza [8]. Recently, infectious agents have also been suggested as possible BP-inducers [6,10]. However, the relationship between infections and BP is not only limited to the infections as possible disease-trigger agents, but they may represent a frequent and severe complication [13], even in consideration of the immunosuppressive effect of therapy, capable of heavily influencing the clinical course and the mortality rate [14].

The disease pathogenesis is linked to the effect of anti-BP180 and anti-BP230 autoantibodies, which may link their respective antigens at the dermal–epidermal basement membrane (BM) and complement activation, so that chemotactic factors are released to attract inflammatory cells able to release proteases, free oxygen radicals, and leukotrienes, such as neutrophils, mast-cells, eosinophils, and macrophages. The consequent inflammation is responsible for local damage, with detachment of the epidermis from the dermis at the BM level [2,14]. The anti-BP180 and BP230 autoantibodies are of IgG isotype (including IgG1 and IgG4 subclasses), but also of IgA and IgE. IgE autoantibodies may be observed in up to 86% of untreated BP patients [15]. This, together with the relevant pathogenetic role of eosinophils [16,17], underlines the tight interrelationship between allergic and autoimmune immunopathogenic mechanisms, which tightly synergize to induce the tissue damage. Recently, the immunopathologic role of the cytokine IL17A has also been identified [18], thus providing the suggestion for a further therapeutic approach, represented by anti-IL17A monoclonal antibodies. However, autoimmunity is not only witnessed by the identification of antibodies of different isotypes recognizing autoantigens, but also by the demonstration of the epitope spreading phenomenon; in fact, the autoantibodies are initially exclusively directed toward the extracellular component NC16A of the BP180 transmembrane autoantigen, whereas, subsequently, autoantibodies directed toward the intracellular component appear [2].

The diagnosis is based on clinical characteristics, which, in the bullous phase, are easy to be recognized, and on laboratory features, such as histology with the demonstration of a linear deposit of IgG and/or C3 at the dermal–epidermal BM by direct immunofluorescence microscopy of a perilesional skin biopsy and the detection of serum autoantibodies against BP180 and/or BP230 [14].

A BP patient in whom multiple bacterial infections have been simultaneously documented with the BP flare is described here.

## 2. Case Report

**Figure 1:**
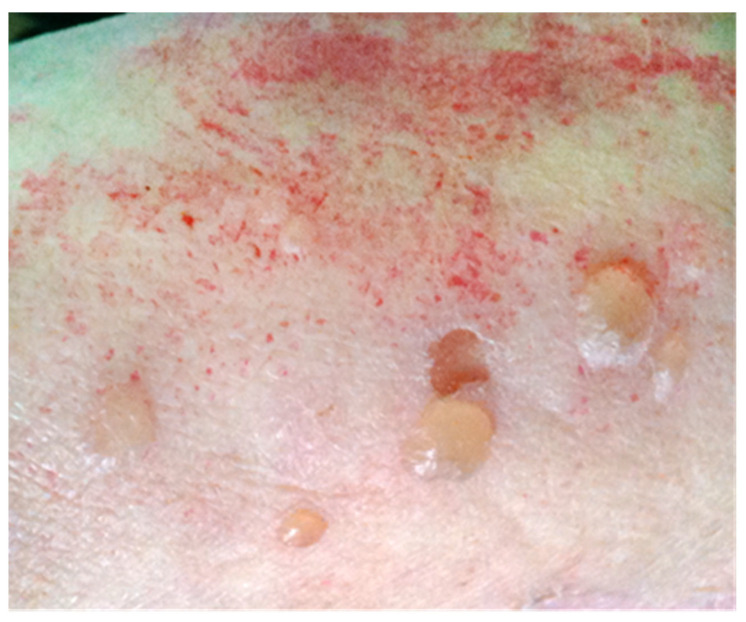
Blistering skin lesions on erythematous base on the right arm.

A 75-year-old woman was admitted to the Clinical Immunology unit of the S. Andrea University Hospital because of an itchy rash characterized by bullous hemorrhagic lesions over an erythematous base, which appeared 6 days before and gradually spread to the entire body, with no mucosal involvement (Figure 1). The condition was also accompanied by fever (38 °C), cognitive impairment, and lack of self-sufficiency.

She had a history of type 2 diabetes, hypertension, and stroke (5 years before), resulting in hemiplegia of the upper and lower right limbs; no history of allergy, autoimmunity, or cancer was referred. The patient had been on furosemide and vildagliptin for one year.

Laboratory tests revealed severe hyponatremia (120 mmol/L), leukocytosis (18,640/µL), moderate anemia (Hb 10 g/dL), and increased C-reactive protein (2.9 mg/dL), whereas the serum protein electrophoresis showed high α2-globulin (0.9 g/dL) and low γ-globulin (0.5 g/dL) levels. Serum immunoglobulin (Ig) values were: IgG 684 mg/dL, IgA 158 mg/dL, IgM 29 mg/dL, and IgE 1,273 IU/mL. Blood cell counts revealed hyper-eosinophilia (3170/µL; normal values < 500/µL); during the over 1-month permanence in the hospital, eosinophils presented a fluctuating trend (median 1132; range 40–3170/µL), with peaks alternating with lower (even normal) values, which were associated with clinical improvement. Serum anti-BP180 IgG was tested by the commercial enzyme-like immunosorbent assay (ELISA, MBL, Nagoja, Japan) and resulted positive (129.7 U/mL, normal values ≤ 9), whereas anti-BP230 resulted negative. Lesions were monitored with the BP Disease Area Index (BPDAI) [19]. The score at diagnosis was 70/120.

Intravenous methyl-prednisolone (40 then 20 mg/day) was administered, and hyponatremia was gradually corrected, whereas furosemide and vildagliptin were discontinued on suspicion of drug responsibility in the BP induction.

**Figure 2:**
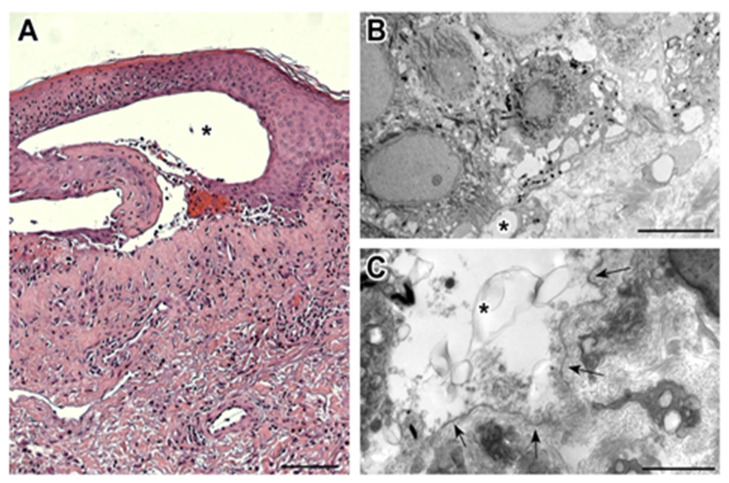
(**A**) The bullous lesion at histopathologic examination is composed of a subepidermal blister (*), with underlying sclerosis of the papillary component of the dermis, dilated capillary vessels, and perivascular chronic lymphocytic infiltrate with an eosinophilic component. A significant actinic elastosis is visible in the reticular dermis. (Hematoxylin/eosin staining; 100× magnification; bar 100 µm). (**B**) Transmission electron microscopy performed on the perilesional skin reveals the presence of small subepidermal blisters (*) without epidermal basal cell damage (uranyl acetate/lead citrate; bar: 5 μm). (**C**) Ultrastructural analysis of lesional skin shows the formation of a blister (*) between the basal keratinocytes and the partially fragmented lamina densa (arrows), which defines the floor of the blister (uranyl acetate/lead citrate; bar: 2 μm).

Histopathological and ultrastructural examination of a perilesional and lesional skin biopsy showed a pattern compatible with BP (Figure 2).

Cultures of the bullous fluid revealed the growth of *Enterococcus faecalis*, and one blood culture was positive for *Staphylococcus aureus*. The urine culture was positive for *Proteus mirabilis* and *Escherichia coli.* The patient underwent antibiotic therapy with intravenous ampicillin (1 g × 2/day for 6 days) followed by piperacillin plus tazobactam (4.5 g × 2/day for 6 days) and, subsequently, linezolid (500 mg × 2/day for 7 days) and meropenem (1 g × 2/day for 5 days), according to antibiogram results. Radio-allergo-sorbent tests (RASTs) for G and V penicillins, as well as for ampicillin, were positive. Skin lesions were locally treated with boric acid solution 3% and eosin solution 2%.

At discharge, the patient was apyretic, had recovered her level of self-sufficiency, and appeared fully oriented. Azathioprine (50 mg/day) was started and intravenous steroid was moved to oral administration (prednisone 25 mg/day). Itching still persisted, whereas, at clinical examination, total skin recovery was observed. Blood tests showed normal levels of leukocytes and reduced hyper-eosinophilia (leukocytes 8960/µL and eosinophils 1057/µL), whereas hypogammaglobulinemia persisted (ɣ-globulins 0.56 g/dL; IgG 490 mg/dL) and C-reactive protein was still increased (4.3 mg/dL). The gradual tapering of the steroid dose up to its interruption was completed in one year, the azathioprine was stopped after two years, whereas the periodical infusion of intravenous immunoglobulins (IVIg, 1 g/kg monthly) was continued for one further year [20]. The patient stopped any treatment five years ago and, in this period, has always been in good health. A brief summary of the clinical and laboratory characteristics of the patient at admission to the hospital and at discharge is reported in Table 1.

## 3. Discussion

In the case described here, BP has been diagnosed on the basis of the criteria needed for a BP diagnosis to be made, which is a compatible clinical picture, histology, even supported by ultrastructural analysis, and the presence of circulating anti-BP180 autoantibodies identified by ELISA. In fact, the anti-BP180 and BP230 hemidesmosomal proteins IgE, IgG1, and IgG4 antibody production [21] have been reported as a BP hallmark. BP180 and BP230 play structural roles in stratified epithelia, and their immune-mediated damage causes a dermo-epidermal cleavage, with resulting blister formation. This pathological sequence involves mast cell degranulation [22,23].

The BP180/BP230 expression in the central nervous system [24] may provide the basis for the BP association with neurological diseases [25]. In the patient described here, the previous stroke, with consequent hemiplegia and bedridden condition, is in line with the well-known BP risk factors. The tight correlation between clinical worsening or improvement and eosinophil counts allows one to hypothesize a pathogenetic role for eosinophils in this specific case, thus suggesting a drug-induced pathogenesis, even though a BP-associated hyper-eosinophilia independent of an allergic state has been described [26].

The association between BP occurrence and both furosemide [27,28] and vildagliptin [29] administration has been reported in the literature. Several BP cases associated with furosemide intake have been described [27,28], and different possible pathogenetic mechanisms have been hypothesized, although formal demonstrations have seldom been achieved [8]. Loop diuretics have been used more frequently in BP-developing patients in a case-control study [30]. Takeishi et al. described a BP case correlated with furosemide intake and sunlight exposure, suggesting that furosemide may act as a photosensitizer [31]. Panayiotou reported two more elderly patients with heart failure, in whom BP appeared with intravenous furosemide intake and resolved after its discontinuation [32]. Vildagliptin belongs to the group of oral dipeptyl peptidase-4 inhibitors (DPP-4is). Several cases of DPP-4is (especially vildagliptin)-induced BP have recently been reported and epidemiological studies have corroborated such an association, even though the pathogenetic process still remains largely unknown [33].

The timing of new lesion onset and the specific IgE test results support a reaction also against beta-lactamic ring antibiotics; in fact, after antibiotic therapy discontinuation, a progressive improvement was observed, without new bullous lesions. However, the clinical improvement may even be interpreted as a consequence of the healing from infections. In fact, the above-reported hypothesized drug-induced pathogenesis does not exclude other possible triggers possibly being involved. Infections have traditionally been considered possible triggers of autoimmunity [34] and, more recently, they have even been suggested as possible BP inducers [8] (Table 2). Human herpesviruses have been found in blister fluid of BP patients [35] and serological association has even been observed [6]. The Torque Teno virus has been selectively found to be associated with BP [36], as well as human immunodeficiency virus (HIV) [37,38,39], hepatitis C virus (HCV) [40], and hepatitis B virus (HBV) [6]. Even coxsackievirus A6 has been reported to be able to induce a BP flare [41]. Moreover, bacterial infections, such as *Helicobacter pylori* [6] and the bacteria involved in erysipelas [42], have also been found to be BP-associated. Finally, even parasites, such as *Toxoplasma gondii* [6] and *Sarcoptes scabiei* [43], have been associated with BP. In the patient described here, the culture of blister fluid was positive for *E. faecalis,* the blood culture was positive for *S. aureus*, and the urine culture was positive for *P. mirabilis* and *E. coli.* All these simultaneous infections were probably determined by the lack of self-sufficiency and humoral immunodepression, documented by the low serum levels of γ-globulins and IgG. In fact, it is well-known that older age is accompanied by immune-senescence [44], with a significant functional reduction of different immune compartments, including the humoral one, with a defective response to vaccines, a reduced number of memory B cells, and reduced levels of circulating immunoglobulins. Infections and chronic inflammatory diseases can act on this predisposing substrate and induce a worsening of the immune system function [45]. The positivity of blood culture for *S. aureus,* a microorganism frequently found associated with erysipelas, which, in turn, has been described as BP-associated, and the positivity of blister fluid for *E. faecalis*, are both suggestive elements for a possible bacterial responsibility in triggering BP. However, the induction of autoimmune reactions by bacteria was known for long time, through the molecular mimicry mechanism, as in rheumatic fever and *Streptococcus β hemolytic group A*, or through the bystander activation [46]. Even the improvement observed after the end of antibiotic therapy and the successful treatment with IVIg, a cross-road between an anti-infective and an immune-modulating treatment, is a further clue to suspect that the sudden burst of the blistering autoimmune disease may have been induced by an infectious trigger, the removal of which was parallel to the clinical BP improvement. Although IVIg has been empirically used at a dose that is half that generally indicated in autoimmune diseases and has recently been demonstrated effective in BP through a randomized study [47], they were highly effective and induced a disease stabilization.

Autoimmune and allergic diseases share important features; one of the potential common elements may be represented by mast cells, which have recently been found to be involved in the development of autoimmune diseases, such as rheumatoid arthritis, type I diabetes, multiple sclerosis, and BP [48]. In BP, mast cells play a key role in neutrophil recruitment [22]. Allergic and autoimmune mechanisms seem to be strictly interwoven in the pathogenesis of the current case and supported by detection of hyper-IgE, hyper-eosinophilia, the presence of anti-G/V penicillins and ampicillin IgE, the detection of autoantibodies to BP180, and indirect signs of BP180-anti-BP180IgG immune complexes at the dermal–epidermal junction as well.

## 4. Conclusions

The patient described here is the first, to the best of our knowledge, in whom bacterial pathogens have been isolated and identified contemporaneously with a BP flare, thus suggesting that bacterial infections may act as BP triggers. In fact, for *H. pylori*, only serology was analyzed, and, for erysipelas, the etiologic agent(s) has(ve) not been identified. The easy BP diagnosis allows one to establish with certainty that the onset of autoimmune disease and bacterial infections is contemporaneous, thus making this case report a clear example of the tight, possibly pathogenetic, relationship between infections and autoimmune diseases. Conversely, the simultaneous positivity of four different bacterial pathogens is a further confirmation that infections are a frequent and severe complication of BP, able to influence prognosis and to increase the mortality [49], in analogy to what may be observed in other autoimmune diseases [50]. In this context, the role of IVIg as an immunomodulating stabilizing agent in a BP case associated with many severe bacterial infections has to be underlined. Moreover, this case is a faithful expression of the synergy between autoimmune and allergic immunopathologic mechanisms in BP, a synergy that may even be observed only in few other autoimmune diseases, such as some cases of vasculitis, of which the anti-neutrophil cytoplasmatic antibody-associated eosinophilic granulomatosis with polyangiitis (formerly Churg–Strauss Syndrome), in which allergic and autoimmune clinical and laboratory manifestations are strictly interwoven, is the most representative example.

## 5. Ethic Statement

The patient provided permission to publish clinical data anonymously.

## Figures and Tables

**Table 1:** Clinical and laboratory patient characteristics at admission to and discharge from the hospital.

Admission	Discharge
*Clinical conditions* Fever 38 °C Diffuse bullous hemorrhagic rash/no mucosal involvement Cognitive impairment/no self-sufficiency	*Clinical Conditions* Apyretic Skin recovered/itching persisted Oriented/self-sufficient
*Cultures* Blood: S. aureus Blister fluid: E. faecalis Urine: P. mirabilis and E. coli	*Cultures* Negative Negative Negative
*Allergy* Hyper-eosinophilia (3170/µL) Hyper-IgE (1273 IU/mL) RAST+ for G/V penicillins and ampicillin	*Allergy* Hyper-eosinophilia (1057/µL) ND ND
*Autoimmunity* Antibodies anti-BP180 (129.7 U/mL)	*Autoimmunity* ND
*Laboratory tests* Leukocytosis (18,640/µL) Moderate anemia (Hb 10 g/dL) Hyper-C-reactive protein (2.9 mg/dL) Hyper-α2-globulinemia (0.9 g/dL) Hypo-ɣ-globulinemia (0.5 g/dL) Hypo-IgG (684 mg/dL) Hyponatremia (120 mmol/L)	*Laboratory tests* Leukocytes (8960/µL) Moderate anemia (Hb 8.7 g/dL) Hyper-C-reactive protein (4.3 mg/dL) Normal α2-globulinemia (0.66 g/dL) Hypo-ɣ-globulinemia (0.56 g/dL) Hypo-IgG (550 mg/dL) Normal natremia (138 mmol/L)
*Biopsy perilesional skin* ***Histopathology*:** subepidermal blister, perivascular lymphocytic infiltrate with eosinophils ***Ultrastructural analysis*:** small subepidermal blisters without epidermal basal cell damage	*Biopsy perilesional skin* ND ND
*Treatment* Furosemide interrupted Vildagliptin interrupted Ampicillin (1 g × 2/day iv for 6 days) Piperacillin/Tazobactam (4.5 g × 2/day iv for 5 days) Linezolid (500 mg × 2/day iv for 7 days) Meropenem (1 g × 2/day iv for 5 days) Hyponatremia gradual correction Methyl-prednisolone (40 then 20 mg/day iv)	*Treatment* Prednisone (25 mg/day for 1 yr) Azathioprine (50 mg/day for 2 yr) IVIg (1 g/kg/month, for 3 yr)

RAST = radio-allergo-sorbent-test; ND = not done; iv = intravenously.

**Table 2:** Infectious agents suspected of acting as triggers of bullous pemphigoid and type of diagnosis of infection.

Microorganisms/Infections	Clinical	Cultural	Microscopic/Molecular	Serological	References
CMV-EBV-HHV6-HHV8			x	x	[6,35]
Torque Teno virus			x		[36]
HIV			x		[37,38,39]
HBV				x	[6]
HCV			x		[40]
Coxsakievirus A6	x				[41]
*Helicobacter pylori*				x	[6]
Erysipelas	x				[42]
*Toxoplasma gondii*				x	[6]
*Sarcoptes scabiei*	x		x		[43]
*Staphylococcus aureus*		Blood			This case
*Enterococcus faecalis*		Blister fluid			This case

CMV = Cytomegalovirus; EBV = Epstein–Barr virus; HHV6/HHV8 = Human Herpesvirus 6/8; HIV = Human Immunodeficiency virus; HBV = Hepatitis B virus; HCV = Hepatitis C virus.

## Data Availability

Sant’Andrea University Hospital, Rome, Italy.
